# Sniffing out olfactory reference syndrome

**DOI:** 10.4102/sajpsychiatry.v23.1016

**Published:** 2017-01-31

**Authors:** Eileen Thomas, Juané Voges, Bonginkosi Chiliza, Dan J. Stein, Christine Lochner

**Affiliations:** 1 Department of Psychiatry, Faculty of Medicine and Health Sciences, Stellenbosch University, South Africa; 2 US/UCT MRC Unit on Anxiety and Stress Disorders, Department of Psychiatry and Mental Health, University of Cape Town, South Africa; 3 US/UCT MRC Unit on Anxiety and Stress Disorders, Department of Psychiatry, Stellenbosch University, South Africa

## Abstract

Olfactory reference syndrome is characterised by the erroneous belief that one emits an unpleasant body odour. This results in significant distress and is often accompanied by repetitive behaviour such as frequent showering in an attempt to camouflage the perceived odour. The body odour concerns may have a delusional quality and do not respond to simple reassurance or counterexample. Herein, we report the case of an olfactory reference disorder (ORD) patient who had received multiple medical interventions and undergone polysurgery prior to an accurate diagnosis being established. ORD may lead to significant disability, yet often goes unrecognised for many years. For many patients, poor insight will contribute to their reluctance to consider psychiatric treatment. This case demonstrated that a multimodal treatment approach comprising judicious medication use, combined with cognitive behavioural therapy, in the context of a therapeutic alliance yielded therapeutic success.

## Background

Olfactory reference syndrome (ORS) is characterised by the erroneous belief that one emits a foul or unpleasant body odour.^1^ This condition is characterised by the belief that others take special notice of the unpleasant odour, prompting repetitive behaviour such as frequent showering or excessive deodorant use to camouflage the perceived odour. As individuals with ORS believe they are the cause of social offence, interpersonal interactions are avoided or else endured with significant distress with the accompaniment of shame and embarrassment.^2^ The body odour concerns may have a delusional quality, that is, they may be fixed and unwavering and the person cannot be dissuaded despite evidence to the contrary or reassurance to the contrary.

The first published descriptions date back to the late 1800s.^3^ Several other case reports and small case-series have since then been published from around the world.^4,5^ Notably, the term ORS was first used in 1971 to describe the consistent phenomenology noted in a large patient case-series.^2^ Here, Pryse-Phillips and his colleagues identified 100 cases of patients who experienced olfactory hallucinations of unpleasant body odour and the concomitant belief of patients that people were reacting negatively towards them because of it.

Despite increasing recognition of the olfactory reference symptomatology, the nosological status of ORS remains unclear. In both the DSM-IV-TR as well as the International Classification of Diseases 10 (ICD), ORS patients with no insight may meet the criteria for a delusional disorder (somatic type). Although ORS was proposed for inclusion in DSM-5 as an independent disorder,^6^ this classification system only mentions ORS in the section on ‘other specified obsessive-compulsive and related disorders’. Specifically, the DSM-5 chapter of Obsessive Compulsive and Related Disorders (OCRD) includes an ‘insight specifier’. The level of insight into the excessiveness or irrationality that individuals with OCD have into their symptoms varies widely, and the intention with the inclusion of this specifier is to improve differential diagnoses by highlighting that an individual who presents with delusional beliefs may warrant a diagnosis of the relevant OCRD, rather than schizophrenia or another psychotic disorder. ORS is currently being considered for inclusion in the upcoming ICD-11.^7^

When patients present with ORS symptoms, a range of differential diagnoses should be considered.^4^ Indeed, this condition can present a diagnostic dilemma in clinical practice. Descriptions of ORS cases may foster further research and promote its recognition by clinicians. In the following report, we describe an interesting ORS case with comorbid depressive symptomatology. This patient was thought worthy of presentation for a number of reasons such as his atypical older age of onset of ORS, and his interesting treatment history which included numerous visits to clinicians from different disciplines and polysurgery. Furthermore, a multimodal approach employing a combination of judicious medication use, combined with cognitive behavioural therapy (CBT), in context of a therapeutic alliance resulted in treatment success.

## Case introduction

A 46-year-old single male was referred for assessment to Consultation Liaison Psychiatry services at a tertiary academic hospital in Cape Town, with a complaint of a bad odour that had been emanating from his body for the past 8 years. No distinct antecedent events were reported prior to the onset of an increasing awareness that people were sniffing and coughing around him. Over the next few months, he came to believe that an unpleasant smell, described as ‘rotting’, was emitting from multiple sites of his body: his nose, throat, axillae, feet and anal area. He noticed his colleagues rubbed their noses when he passed by, purposefully opened windows or turned away in disgust when he was in their presence. Remarks by others of smells were interpreted as pertaining to him. In an attempt to eliminate and minimise the odour, a daily ritual of time-consuming repetitive behaviours ensued, including frequent laundering and change of clothes, frequent deodorant use and alteration of his diet.

Over the last few years, he had sought relief from several medical specialists, including dermatologists, gastroenterologists, and ear nose and throat specialists. A number of surgeries, including turbinoplasties, and a tonsillectomy were performed, yet the odour and the resulting distress persisted. Socially isolating himself, he resided with his brother, for whom he felt he was a source of great shame and embarrassment. Previous hobbies, such as hiking had been abandoned, as he was concerned about how the odour affected and offended others. He had passed up on several job promotions, and avoided dating and other social interactions. Using public transport was described as the most distressing daily event. He managed the discomfort by sitting afar from other people, switching train carriages to avoid notice of the supposed odour and listening to music on his earphones as a distraction.

Developmental history of this individual was remarkable for childhood enuresis. He is the eldest of three siblings and describes his childhood as lonely. His father, a man with alcohol dependence, would become verbally abusive when inebriated. After graduating from high school, he completed 4 years of non-combative military duty during the time of political unrest in South Africa. He has been permanently employed as a security surveillance agent for the past 12 years. The patient had no history of past or current illicit substance use. He had no children and no previous marriages. There is no family history of psychiatric illness. Describing himself as shy, he appeared to be otherwise well-adjusted premorbidly.

## Assessment

On initial assessment, mental state examination revealed a healthy looking male patient who appeared his stated age. He was alert, orientated and an accessible historian. He was aloof, but not strikingly apathetic. He presented with a depressed mood, with suicidal ideation and had a low-volume aprosodic speech. His thought form was coherent and goal directed. Thought content included an unchallengeable belief that his body was emanating a foul odour and that others were also aware of this. Additionally, he reported intermittently smelling the odour. There were no other psychotic or obsessive-compulsive phenomena. He claimed no discomfort with his physical appearance. He attributed his depressive symptoms to the odour concerns and the resulting social isolation he experienced. On clinical examination, there was no odour and his physical examination and all relevant special investigations, including brain and sinus imaging, were within normal limits.

The Brown Assessment of Beliefs Scale (BABS)^8^ was administered to assess insight into his delusional beliefs, with a composite BABS score of 20 recorded, correlating with a poor degree of insight. His score on the Beck Depressive Inventory II (BDI-II)^9^ was high (BDI = 30), suggesting severe depressive symptomatology. A diagnosis of ORS and comorbid depression was made.

## Course of treatment and assessment of progress

Our patient discontinued prior antipsychotic drug treatment, owing to reported tolerability issues. He consequently expressed reluctance to attempt another antipsychotic drug trial. Thus, the decision was made to prescribe fluoxetine 20 mg owing to marked comorbid depressive symptomatology. A structured CBT course was offered in tandem. The patient agreed to both. Weekly CBT sessions, based on an individualised formulation, commenced in a collaborative, non-didactic and graded fashion. The treatment goal was a lessening of the distress and impaired functioning that was consequent of the psychotic symptom experience. This included identifying the biased appraisal processes that were maintaining the distressing beliefs and assisting the patient to generate alternate and more helpful, healthy and balanced explanations and thoughts. Behavioural strategies employed included relaxation, graded exposure, activity scheduling, distraction and problem solving.

The early CBT sessions were used to establish a therapeutic alliance that modelled a climate of unconditional acceptance, rather than focusing directly on challenging his odour-beliefs. The therapist maintained a non-judgemental, inquisitive stance towards his difficulties and perceptions of others. This positive regard was helpful to circumvent negative self-views that had hindered previous engagement.

Given the delusional intensity with which smell-related beliefs were held, we first worked on the less firmly held beliefs. Traditional cognitive techniques were employed, including identifying biased appraisal processes that were maintaining the negative beliefs regarding self and assisting him to generate alternate and more helpful, healthy and balanced explanations and thoughts. Behavioural strategies were employed to address social avoidant behaviour and time-consuming compensatory behaviour. The sequence of prefacing behavioural with cognitive strategies was done to lessen our patient’s expressed fears and misconceptions around exposure exercises. Breathing and relaxation techniques improved his ability to manage his anxiety in social settings. Positive activity scheduling and exposure techniques were graded according to situations our patient felt able to engage in. For example; he was encouraged to ride his bicycle on his own, before attending a sport recreation centre. This increased his sense of mastery and tolerance for discomfort in anxiety-evoking situations.

At 6-week follow-up, an increase in fluoxetine-dosage to 40 mg was deemed necessary, and our patient responded positively with decreased depressive symptomatology as well as time spent engaging in safety behaviours. At 3-month follow-up, delusional beliefs and preoccupations remained highly salient, however, with continued intermittent olfactory hallucinations. Risperidone at initial dosage of 1 mg was suggested as an augmentation drug and was accepted by the patient.

At 5-month follow-up, depressive symptoms had abated (BDI = 4) with reported improvement in socio-occupational functioning. This was evidenced by renewed participation in hobbies such as cycling and hiking, improved social interaction with family and co-workers, and improved work-appraisal reports. Furthermore, he enrolled for a long-desired evening security diploma course and expressed interest in dating.

Although the referential thoughts about odour still continued, they were much less frequent and intense and he was able to express doubt regarding the veracity of his perceptions. This was reflected by his score of 13 on the BABS. He now only infrequently experienced the foul odour himself and the duration of time-consuming safety behaviours (such as cleaning, washing and avoiding) have lessened markedly.

## Complicating factors

On initial consultation, our patient expressed ambivalence and noted that he felt the reason the odour persisted was owing to his inability to adequately relay his symptoms to other health care providers. He consequently also requested referrals to dermatologists and ear, nose and throat specialists. The first step in the successful treatment of this patient involved establishing a therapeutic alliance, which allowed the clinician to effectively engage the patient in treatment, despite the presence of firmly held beliefs and shame associated with the perceived odour.

## Treatment implications of the case

Similar to other case reports,^5,10^ our patient only presented to our psychiatric services after a long period of suffering. He had first consulted numerous medical and surgical specialities for the perceived odour spanning an 8-year period in total. These resulted in various medical treatments such as recurrent antibiotic trials, and allergy treatment including corticosteroids being employed, yet the symptoms persisted. Subsequently various surgical procedures, including two turbinoplasties and a tonsillectomy were performed, again proving to be ineffective.

Despite its long history, the prevalence of ORS is not well established, estimated to range between 0.5% and 2.1%.^6^ It is possible that the prevalence of ORS is underestimated given that patients may be embarrassed about these symptoms. Patients may therefore not specifically inform clinicians regarding their persistent concerns about the perceived body odour unless relevant screening questions are employed.^1^

Our patient presented with acute onset of faulty odour-perceptions which began at 38 years of age. ORS symptoms are most often reported as beginning when patients are in their mid- twenties,^2,11^ with other reports suggesting an even earlier onset, that is, during puberty or adolescence.^12^ Despite our patient’s later age of onset, the clinical symptoms experienced were consistent with other reported cases in the literature with regard to the referential thinking, time-consuming repetitive behavioural routines, significant interpersonal avoidance and evidence of clinically significant distress and impairment.^12^ Our patient reported intermittently actually smelling the odour, which likely contributed to the high BABS score.

ORS shares overlapping phenomenological features with several other psychiatric disorders, including delusional disorder, somatic type, which is characterised by prominent delusions involving bodily functions or sensations. Some individuals with ORS may hold their odour-beliefs with absent insight/delusional conviction (thus, they are completely convinced that their view of their perceived smell is accurate). In both conditions, patients may present with referential thinking related to bodily concerns. Delusional disorder can however be differentiated from ORS by a lack of key characteristic features including engagement in time-consuming, repetitive behaviours aimed at checking, eliminating or camouflaging the odour as demonstrated in this case.

In terms of comorbidity, major depressive disorder is the most common disorder described in ORS literature.^12^ Depressive symptomatology is generally considered to be secondary to ORS. In Pryse-Phillips’s patient–case series, depressive symptoms tended to be severe. Our patient similarly presented with marked depressive symptomatology and in addition endorsed suicidal ideation.

The ORS treatment literature remains limited, consisting largely of case reports and small case-series. To our knowledge, no randomised controlled treatment trials exist. Until now, treatment agents for ORS have included antipsychotics, antidepressants, combinations of antipsychotics and antidepressants, and psychotherapy. However, given the relative paucity of current treatment data, no concrete or specific conclusions can be made regarding the treatment of ORS, and the best evidence for treatment is derived from the available literature.

It can be particularly challenging to engage delusional patients in treatment, as they are often doubtful whether psychiatric treatment can really help them. However, even patients who maintain that they have an actual physical problem can agree that they are suffering and have a poor quality of life. A mutual goal of decreasing their preoccupation and improving their functioning and quality of life may facilitate engagement in treatment. Providing psycho-education about ORS and conveying that appropriate treatment is likely to improve their symptoms and quality of life are vital.

As with all conditions, it is important to take patients’ concerns seriously by empathising with their suffering. From a cognitive-behavioural perspective, it may be helpful for clinicians to view delusions as the most compelling explanation that the person has arrived at on the basis of previous childhood and adult events, knowledge, emotional states, memories, personality and reasoning processes. In our ORD case, the primary treatment focus was to lessen the distress and functional impairment that had resulted from the odour preoccupation, rather than convince the patient of the falseness of his beliefs. CBT proved invaluable in not only engaging him as an active participant in treatment but also in addressing negative cognitions regarding long-term medication use. The principles of CBT used here are similar to those used in body dysmorphic disorder (BDD) patients with absent insight/delusional beliefs.^13^

It is noteworthy that this patient did not demonstrate remission on antidepressants alone, but that he did respond to augmentation of a selective serotonin reuptake inhibitor (SSRI) with a low-dose atypical antipsychotic. Similarly, it is noteworthy that a form of CBT similar to that used in BDD with absent insight/delusional beliefs appeared useful. Important treatment considerations for this patient included the longstanding nature of his symptoms, the veracity of his delusional beliefs, prior treatment response, treatment availability and comorbidity profile. An SSRI agent was commenced at a low dosage and titrated slowly to maximum tolerable dose. The prescription of a moderate-dose SSRI resulted in lessening of his depressive as well as obsessional/compulsive symptomatology. SSRI use, however, did not improve the delusionality with which the smell-related beliefs were held and these features were assessed as being particularly distressing to our patient. In order to optimise treatment, adherence, efficacy and tolerability, a decision was made to augment treatment with an antipsychotic agent instead of attempting further SSRI dose increases/drug switches. These anecdotal treatment observations are consistent with some previous reports in the literature, as well as with the conceptualisation of ORD as a disorder independent of delusional disorder.

## Recommendations to clinicians and students

This case highlights how clinicians must be prepared to cast their diagnostic net widely, not settling for the obvious, frequently incorrect diagnoses. As demonstrated in this case report, ORS may lead to significant disability, yet often goes unrecognised for many years. Because patients usually do not spontaneously report their symptoms and frequently have poor insight into their condition, clinicians have to elicit symptoms of the disorder with relevant screening questions. For many patients, poor insight will contribute to their reluctance to consider and engage in psychiatric treatment.

The literature regarding ORS treatment remains limited. Whether adding CBT to pharmacotherapy would be beneficial for someone with primary ORS has not been studied systematically. In our case, a multimodal treatment approach comprising a moderate dose of an SSRI with low dose of an atypical antipsychotic, combined with CBT in the context of a therapeutic alliance yielded therapeutic success.

ORS remains a poorly defined clinical entity. Incorporation of ORS into medical classification systems such as the ICD-11 may encourage appropriate diagnosis and act as an impetus to future research. Continued liaison amongst medical specialists will ensure that patients with ORS receive timely and appropriate referral to psychiatric services and avoid unnecessary surgical or medical interventions and investigations. Given the distress and impairment associated with this disorder, improved diagnosis and more research on its pathogenesis and treatment is warranted.
